# l-Carnitine conjugated chitosan-stearic acid polymeric micelles for improving the oral bioavailability of paclitaxel

**DOI:** 10.1080/10717544.2020.1748762

**Published:** 2020-04-20

**Authors:** Tan Yang, Jianfang Feng, Qian Zhang, Wei Wu, Hailan Mo, Lanzhen Huang, Wei Zhang

**Affiliations:** a Department of Pharmacy, Guilin Medical University, Guilin, PR China;; b Department of Pharmacy, Guangxi University of Chinese Medicine, Nanning, PR China

**Keywords:** Micelle, paclitaxel, l-carnitine, oral bioavailability, chitosan

## Abstract

A delivery system based on l-carnitine (LC) conjugated chitosan (CS)-stearic acid polymeric micelles has been developed for improving the oral bioavailability of paclitaxel (PTX) through targeting intestinal organic cation/carnitine transporter 2 (OCTN2). Stearic acid grafted chitosan (CS-SA), as micelle skeleton material, was synthesized by 1-ethyl-3-(3-dimethylaminopropyl) carbodiimide (EDC)-mediated coupling reaction. The PTX-loaded micelles were prepared by solvent evaporation-hydration method, and the ligand LC was conjugated onto the micelle surface by anchoring its derivative stearoyl group to the lipophilic core of micelle. The modified polymeric micelles showed regular spherical shapes with small particle size of 157.1 ± 5.2 nm and high drug loading capacity of 15.96 ± 0.20 wt%, and the micelle stability in water was supported by low critical micelle concentration of 14.31 ± 0.21 μg/ml. The drug-loaded micelles presented a slow and incomplete *in vitro* release, and the pharmacokinetic studies indicated the micelle carriers increased the relative bioavailability of PTX to 165.8% against the commercial formulation. The enhancement effect on intestinal absorption was also confirmed by the intracellular uptake of Caco-2 cells. The proposed micelle carrier system manifested a prospective tool for oral drug delivery.

## 1. Introduction

Although the study on improving the oral absorption of poorly soluble drug, especially anticancer chemical drug, has been going on for years, an eventual solution strategy was still owed the topic. Since an increasing number of anticancer compounds, including doxorubicin, docetaxel, catechin, vinblastine, chrysin, and baicalein, were faced with the issue of low oral bioavailability, the injection administration had to be introduced despite of poor compliance or side effect. Paclitaxel (PTX), an anti-microtubule drug widely used in various cancers, classified as biopharmaceutic classification system (BCS) class IV drug, is mainly formulated into the commercial injection Taxol^TM^ because of its low solubility and low permeability. In the formulation, the excipient Cremophor EL is known to produce severe side effects, including hypersensitivity, nephrotoxicity, and neurotoxicity (Xu et al., 2015). Although the alternative formulations have been developed, such as Abraxane^®^ (PTX protein-bound particles as an injectable suspension) and Lipusu^®^ (PTX liposome for injection) (Zhao et al., 2017), the patients have to pay high price for them. Therefore, there is an urgent need to develop high-efficacy, low-toxicity, and low-cost oral PTX products.

For the past few years, a large number of efforts have been paid to developing nanometer delivery system for improving the oral absorption of insoluble antitumor drugs, including micelles (Yao et al., 2017; Ma & Williams, 2018), liposomes (He et al., 2016; Hussain et al., 2016; Luo et al., 2016), nanogels (Voeikov et al., 2017), nanoparticles (Choi et al., 2014; Kang et al., 2015; Qi et al., 2016), and nanocrystals (Choi & Park, 2016, 2017). Polymeric micelle, as an ideal carrier of hydrophobic drugs, received increasing attentions due to their excellent properties, e.g. small particle size, high drug loading (DL) capacity, enhanced bioavailability, and depressed side effects (He & Yin, 2017). Amphiphilic chitosan (CS) derivative micelles, composed of modified natural polysaccharide, have been attracting interests in the past decades, due to the bioadhesiveness, biocompatible, and biodegradable of CS. The micelles can be facilely obtained by self-assembly in aqueous environment, and the micelle outer shell exposing to aqueous phase would protect the carrier system from inactivation in biological media while the inner core embedded in the hydrophilic palisade is used as micro-storages for insoluble drugs (Wang et al., 2015).

The impact of intestinal transporters on drug oral absorption has become increasingly evident, and many interests have focused on the role of drug transporters in gastrointestinal tract. The drug carrier systems designed to target the intestinal transporters, including P-glycoprotein (P-gp), oligopeptide transporter (PEPT), monocarboxylate transporter (MCT), sodium-dependent vitamin C transporter 1 (SVCT1), and organic anion transporting polypeptide (OATP), have become one of the most prevalent trends in drug delivery (Ahmad et al., 2019; Wang et al., 2019). The novel organic cation transporter 2 (OCTN2) is located in the apical and basolateral membranes of intestinal cells. l-Carnitine (LC), as the endogenic substrate of OCTN2, is an utilizable ligand for OCTN2-targeted carrier system improving the intestinal absorption of drug. OCTN2-mediated nanoparticles and prodrugs for oral delivery of therapeutic drugs have been developed, and the role of the intestinal transporter in enhancing drug oral absorption was confirmed (Wang et al., 2017).

**Figure 1.**
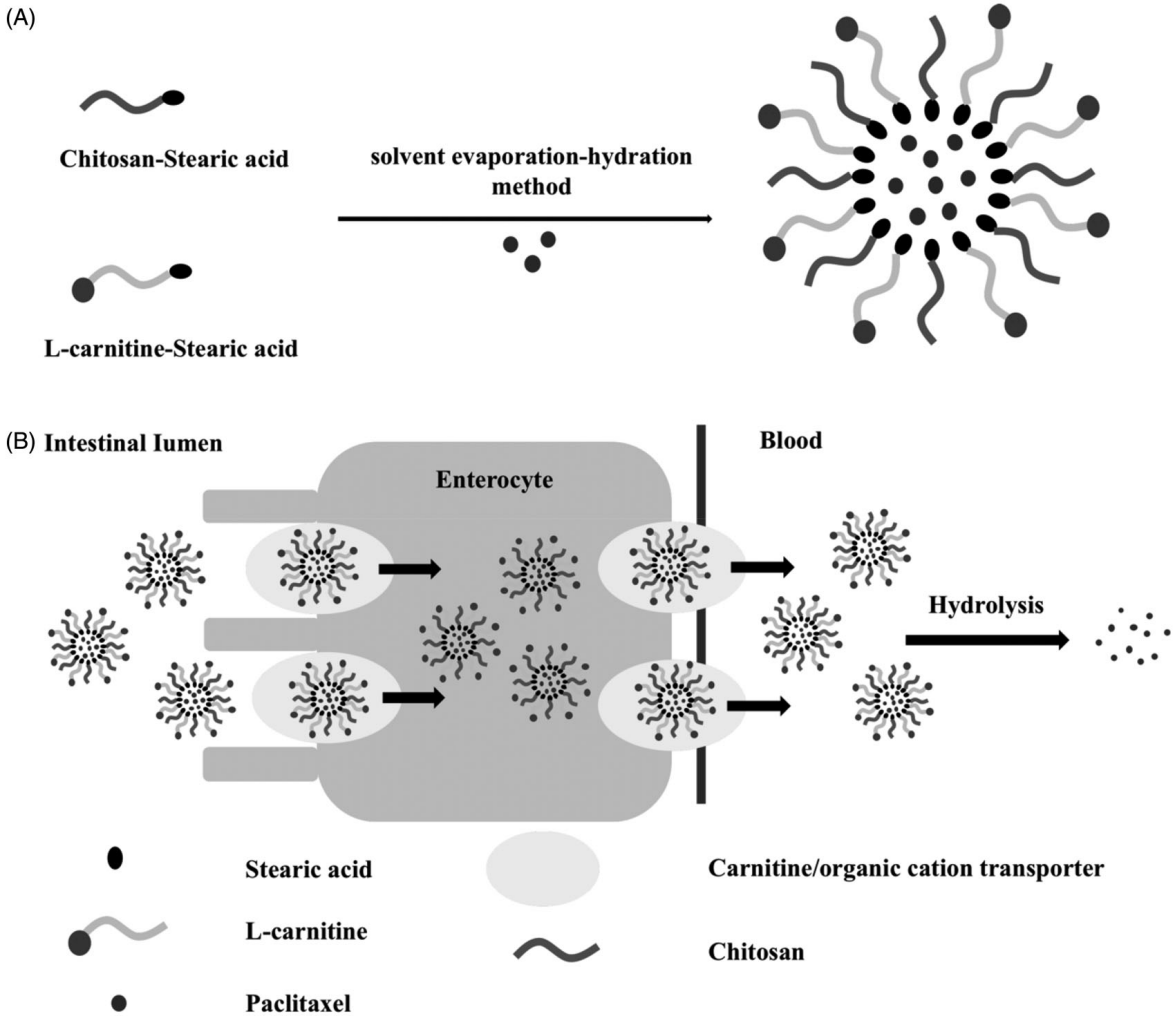
The enhanced effect of LC-SA/CS-SA micelle on the oral absorption of PTX. (A) The preparation of PTX-loaded LC-SA/CS-SA micelles; (B) the OCTN2-mediated absorption mechanism of PTX encapsulated by LC-SA/CS-SA micelles.

In the present study, a delivery system based on LC conjugated chitosan-stearic acid (CS-SA) micelle was designed for improving the oral absorption of PTX as model drug. The micelle skeleton CS-SA utilized the bioadhesiveness of the outer shell CS for the synergistic enhancement of intestinal absorption, and the hydrophobic core of SA for the high loading capacity of lipophilic drug. The ligand LC was anchored onto the surface of micelle via the stearoyl group of its derivative LC-stearic acid (LC-SA), and the PTX-encapsulated LC-SA/CS-SA micelles would pass through enterocytes via OCTN2-mediated transportation for improving the intestinal absorption of PTX. The structure of the modified drug-loaded micelle and the intestinal transportation process can be summarized in Figure 1. In this hypothesis, the enhanced effect of LC-SA/CS-SA micelle on the oral absorption of the model drug would be confirmed, and a series of evidence would be provided for the confirmation of the OCTN2-mediated mechanism and intact micelle transportation mode. To our best knowledge, there was no previous report on OCTN2-mediated micelle for enhancing the intestinal absorption of insoluble drug.

## 2. Materials and methods

### 2.1. Materials

Chitosan (molecular weight 3–6 kDa, deacetylation degree 93.1%) was obtained from Yuhuan Marine Biochemistry Co., Ltd. (Yuhuan, China). LC-SA was purchased from Shanghai Luzhen Biotechnology Co., Ltd. (Shanghai, China). N-ethyl-N′-(3-dimethylaminopropyl) carbodiimide hydrochloride (EDC) and LC were purchased from Aladdin Reagent Database Inc. (Shanghai, China). Pyrene was obtained from Aldrich Chemical Co., Ltd. (Milwaukee, WI). TRITC-phalloidin was obtained from the AAT Bioquest Co. Ltd. (Shanghai, China). 4′,6-Diamidino-2-phenylindole (DAPI) and Hank’s balanced salt solution (HBSS) were obtained from the Thermo Fisher Scientific Co. Ltd. (Shanghai, China). All other reagents and solvents were of analytical grade and used without further purification. Twenty female and male Sprague-Dawley (SD) rats weighing 220 ± 20 g (*n* = 5 for each group) were fasted overnight before oral gavage but had free access to water, and then divided randomly into four groups. SD rats were purchased from the Silaikejingda Corporation (Changsha, China). All animal studies were carried out in accordance with the Guidelines for Animal Experimentation of Guilin Medical University and the protocol approved by the Animal Ethics Committee of the institution, and complied with the U.K. Animals (Scientific Procedures) Act, 1986 and associated guidelines, EU Directive 2010/63/EU for animal experiments. Caco-2 cells were purchased from the Type Culture Collection of the Chinese Academy of Sciences (Shanghai, China).

Minimum essential medium (MEM), glutamax, non-essential amino acids, and sodium pyruvate 100 mM solution were purchased from Invitrogen (Carlsbad, CA). Fetal bovine serum (FBS) was purchased from Gibco (Mulgrave, Australia).

### 2.2. Synthesis of CS-SA

**Figure 2.**
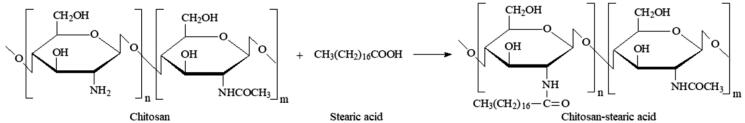
Synthetic route of CS-SA.

According to the previous reports (Xie et al., 2012), CS-SA was synthesized by the EDC-mediated coupling reaction between carboxyl group of SA and amine group of CS as described in Figure 2. Accurate 2.5 mmol of SA and 25 mmol of EDC were dissolved in 80 ml of ethanol while stirring in a water bath at 60 °C for 30 minutes, and 1 g of CS (Mw 3–6 kDa) was dispersed homogeneously in 120 ml of purified water at 80 °C. The ethanol solution was added dropwise into the CS suspension and stirred for five-hour reaction. The residual ethanol was removed by rotary evaporation, and the crude CS-SA was dialyzed against purified water using dialysis bag with molecular mass cutoff of 8–14 kDa. The dialyzed suspension was freeze-dried and washed for four hours with ethanol by Soxhlet extraction to remove the residual SA, and the pure CS-SA can be obtained by further freeze-drying.

### 2.3. Preparation of l-carnitine conjugated CS-SA micelles

The drug-loaded micelles were prepared by solvent evaporation-hydration method. Briefly, 2.3 mg of PTX was dissolved in 2.3 ml of methanol, and 18 mg of CS-SA and 2 mg of LC-SA were dissolved in 30 ml of purified water. The PTX solution was added dropwise into the LC-SA/CS-SA solution. The mixed solution was sonicated for 15 min (per 3 s working time with 2 s break time) at 250 W in an ice bath using ultrasonic cell disruptor to obtain a crude micelle solution. The solvent was removed by rotary evaporation and a transparent thin film formed on the wall of the rotary flask. The resulting film was redispersed in 30 ml of purified water by sonication for 15 min at 45 °C, and the drug-loaded micelle was obtained.

### 2.4. Preparation and characterization of micelles

#### 2.4.1. Nuclear magnetic resonance (NMR) and Fourier transform-infrared (FT-IR) spectroscopy

The molecular structure of the synthesized CS-SA was confirmed by NMR and FT-IR. CS, CS-SA, and LC-SA were dissolved or dispersed in D_2_O, and SA in CDCl_3_ for ^1^H NMR measurements. The substitution degree of CS-SA copolymer was determined by the ^1^H NMR spectrum according to the procedure in the published works (Alani et al., 2010; Wang et al., 2014). The FT-IR spectra of these materials were collected in the region of 400–4000 cm^−1^ in thin KBr pellets.

#### 2.4.2. Particle size and zeta potential

The particle size distribution and zeta potential of PTX-loaded LC-SA/CS-SA micelles were determined using Zeta Potential/Particle Size analyzer (Nano-ZS90, Malvern Instruments Ltd, Malvern, UK). Each sample was measured in triplicate, and the average values were calculated.

#### 2.4.3. Encapsulation efficiency (EE) and DL capacity

The amount of PTX encapsulated in micelles was measured by high-performance liquid chromatography (HPLC, LC-20A, Shimadzu Corporation, Kyoto, Japan). The PTX-loaded micelle solution was diluted 20-fold with methanol and sonicated to destroy the micelle and release the drug using ultrasonic cell disruptor. The obtained suspension was centrifuged at 12,000 rpm and the drug in the resulting supernatant was determined by HPLC, of which the setting condition can be described as follows: Wondacract ODS-2 column (250 mm × 4.6 mm, 5 µm); Mobile phase: methanol:water (75:25, v/v); flow rate: 1 ml/min; detection wavelength: 229 nm. The EE and DL of the micelle can be calculated as follows:

DL (%)=(weight of PTX in micelles/weight of PTX−loaded micelles)×100%

EE (%)=(weight of PTX in micelles/weight of total PTX added into formulation)×100%.

#### 2.4.4. Morphological analysis

The morphology of the PTX-loaded micelle was observed by transmission electron microscope (TEM, HT7700, HITACHI, Tokyo, Japan). The micelle solution was dropped on a transparent carbon film supported by copper grid, air-dried at room temperature, and stained with phosphotungstic acid solution (2%, w/v) for test.

#### 2.4.5. X-ray diffraction (XRD)

The polymorphic status of PTX loaded in LC-SA/CS-SA micelles was analyzed using X-ray diffractometer (SmartLab9, Rigaku Corporation, Tokyo, Japan). The micelle solution was freeze-dried and the corresponding solid-state powder was obtained for test. The tested samples, including pure PTX crystal, blank LC-SA/CS-SA micelle without PTX, PTX-loaded LC-SA/CS-SA micelle and simple physical mixture of PTX and blank LC-SA/CS-SA micelle, were scanned at 6°/min from 5° to 50° of diffraction angle (2*θ*) at 40 kV with 150 mA Cu-Kα radiation (Ciolacu et al., 2011).

### 2.5. Critical micelle concentration (CMC)

The CMC of LC-SA/CS-SA was measured using pyrene as a fluorescence probe to evaluate their abilities of micelle formation and anti-dilution in aqueous solution. In brief, a series of drug-free LC-SA/CS-SA micelle solution with various micelle concentrations from 0.001 to 0.5 mg/ml were prepared, and 1 ml of pyrene-in-acetone solution (6 × 10^−5^ M) was added into the micelle solutions respectively and sonicated for dissolution to obtain the final pyrene concentration of 6 × 10^−7^ M. The pre-prepared solutions were equilibrated overnight at 28 °C, and the emission spectra of the solutions from 350 nm to 500 nm were recorded at an excitation wavelength of 337 nm by fluorescence spectrophotometer (RF-5301PC, Shimadzu Corporation, Kyoto, Japan). The CMC value was defined as the crosspoint of the two regression lines based on the intensity ratios of first peak at 373 nm to third peak at 384 nm vs. the concentrations of micelle material (Dong et al., 2017; Yang et al., 2017; Yin et al., 2017).

### 2.6. *In vitro* release studies

To evaluate the *in vitro* release behavior of the drug-loaded micelles, 2 ml of PTX-loaded LC-SA/CS-SA micelle solution, PTX-loaded CS-SA micelle solution and Taxol^TM^, were placed in a dialysis bag (8–14 kDa cutoff; Shanghai Green Bird Technology Co., Ltd., Shanghai, China) against 50 ml of phosphate buffer (pH 6.8, containing 2% Cremophor EL (w/v)) as release medium at 37 °C. At given intervals, 1 ml of the medium was pipetted out for test and an identical volume of fresh medium was supplemented, and the PTX in samples was detected by HPLC (Li et al., 2012). Each formulation was carried out in triplicate.

### 2.7. Pharmacokinetic studies

Twenty female and male SD rats weighing 220 ± 20 g were divided randomly into four groups (*n* = 5 for each group), and fasted overnight before oral gavage but had free access to drinking water. Groups 1–4 were administrated by gavage with Taxol^TM^, PTX-loaded CS-SA micelle solution, PTX-loaded LC-SA/CS-SA micelle plus LC, and PTX-loaded LC-SA/CS-SA micelle solution respectively as per the PTX dose of 30 mg/kg. Blood samples were collected into heparinized tubes at predetermined time points (0.25, 0.5, 0.75, 1, 1.5, 2, 4, 6, 8, 10, 12, and 24 h) and centrifuged at 3500 rpm for 10 min to obtain the plasma. One hundred microliters of plasma sample was pipetted into Eppendorf tube with the addition of 1 ml of ether, followed by vortex and centrifuging at 3500 rpm for 5 min. The supernatant ether was pipetted into another tube and dried by nitrogen, and then re-dissolved with 100 μl of methanol for test. PTX in plasma was detected under the same chromatographic condition as in section 2.4.3 with an extraction recovery rate of more than 95% and within-day or between-day RSDs of less than 6%, and the chromatographic peak of drug exhibited a baseline separation from plasma matrices. The pharmacokinetic parameters were calculated by DAS 2.0 software (BioGuider Co., Shanghai, China).

### 2.8. *In vitro* cellular uptake studies

The *in vitro* cellular uptake of the micelles by Caco-2 cells was evaluated with coumarin-6 as a fluorescence probe. Caco-2 cells were seeded in six-well plates at a density of 2 × 10^5^ cells/well and cultured for 48 h. When the cells proliferated to cover 50% of the well-bottom area, the culture mediums were renewed with the fresh mediums containing coumarin-6, coumarin-6-loaded CS-SA micelles, coumarin-6-loaded LC-SA/CS-SA micelles plus LC, and coumarin-6-loaded LC-SA/CS-SA micelles respectively and incubated at 37 °C for 3 h. The cells were lightly rinsed with cold HBSS to terminate the uptake process, and fixed with 4% paraformaldehyde. For the qualitative uptake, the cells were stained sequentially with TRITC-phalloidin for cytoskeleton and DAPI for cell nucleus. The cellular uptake profiles were observed and compared by fluorescence microscopy (Axio Imager Z2, Carl Zeiss Group Co. Ltd., Jena, Germany) (Kou et al., 2017). For the quantitative uptake, the content of coumarin-6 in the cells was determined by fluorescence/visible microplate reader (Infinite M200 Pro NanoQuant, Tecan Co. Ltd., Männedorf, Switzerland) (excitation: 466 nm; emission: 504 nm). The protein content of the cells absorbing coumarin-6 was determined by bicinchoninic acid (BCA) method using BCA kit (P0010S, Beyotime Biotechnology Co. Ltd., Shanghai, China) as per the procedure described in the manual of the kit. The uptake levels of the four preparations were calculated and assessed as per the amount of coumarin-6 (μg) per unit protein amount (mg).

## 3. Results and discussion

### 3.1. Synthesis and characterization of CS-SA

The micelle skeleton CS-SA was synthesized by EDC-mediated amido formation between carboxyl group of SA and amine group of CS. Since the carboxyl group was activated by EDC to promote the conjugation to the amine group of CS, the reaction would be accelerated. The molecular weight of CS, as an important factor affecting the reaction yield, was trialed, including Mw 30k, 10k, 3–6k, and 2k. Since CS with high molecular weight was poorly soluble in water and the low was hard to obtain amphiphilic molecule due to its excessive water-solubility, the target molecular weight 3–6k was chosen due to the higher reaction yield of amphiphilic CS-SA.

**Figure 3.**
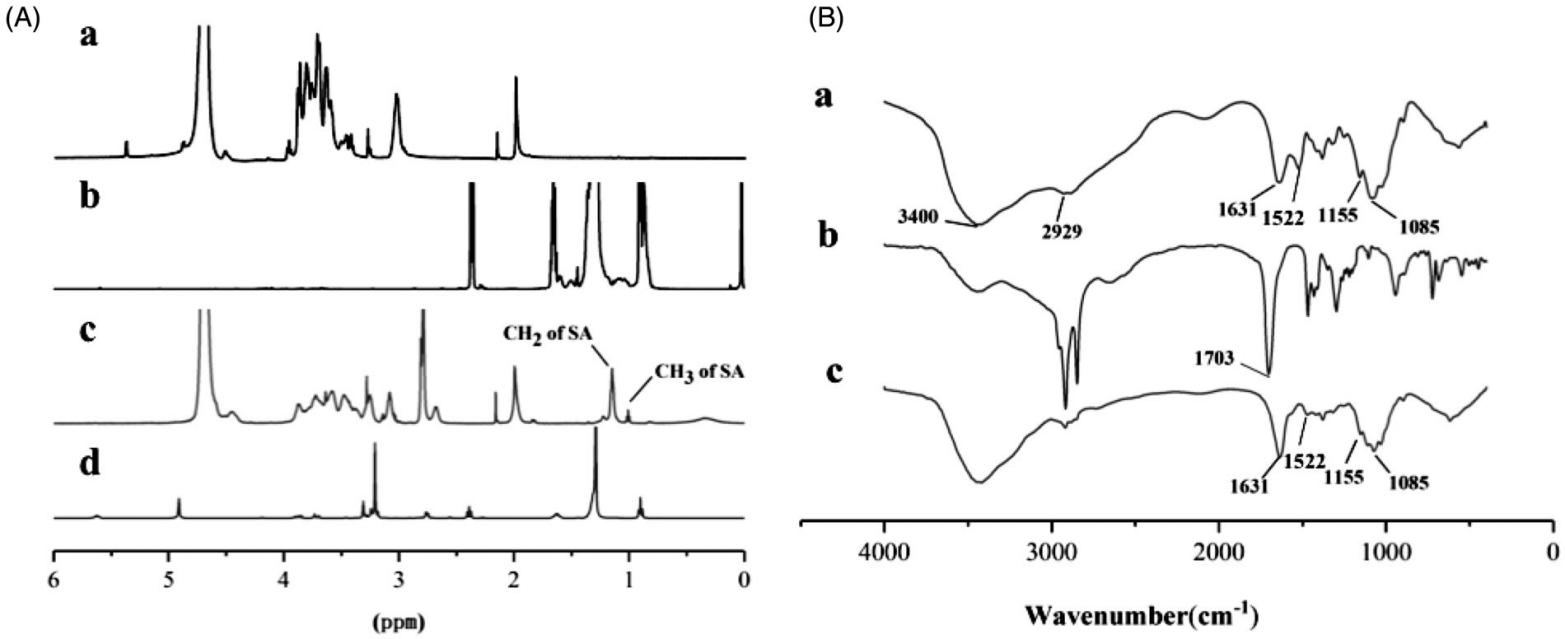
(A) ^1^H NMR spectra of CS (a), SA (b), CS-SA (c), and LC-SA (d); (B) FT-IR spectra of CS (a), SA (b), and CS-SA (c).

The molecular structure of the reaction product was identified by ^1^H NMR and FT-IR. The ^1^H NMR spectra of CS, SA, CS-SA, and LC-SA are shown in Figure 3(A). The typical peaks of CS in the ranging from 3.27 ppm to 3.87 ppm can be assigned to the H-3, H-4, H-5, H-6, and H-6′ of amino glucose unit. According to previous report (Hu et al., 2006), the chemical shifts at ∼0.9 ppm and ∼1.0 ppm in the spectrum of CS-SA can be assigned to the hydrogen on the methyl and methylene of stearoyl group, respectively, which can also be found in the SA. All the other characteristic peaks of CS can be observed in the spectrum of CS-SA, suggesting the successful synthesis of CS-SA. The amino substitution degree of CS-SA was measured to be approximately 57.9%±1.08% by the ^1^H NMR spectrum. The derivative LC-SA showed the characteristic peaks at 3.3 ppm assigned to the hydrogen on trimethyl amino group, which was the recognition site of OCTN2 (Kou et al., 2017).

The FT-IR spectra of CS, SA, and CS-SA are collected in Figure 3(B). A series of absorption peaks, including 3400 cm^−1^, 2929 cm^−1^, and 1631 cm^−1^ assigned to the stretching vibrations of –OH, –CH_3_, and C=O, respectively, can be found in the spectra of CS and CS-SA. The typical CS peaks at 1155 cm^−1^ and 1085 cm^−1^ assigned to the stretching vibrations of C–O (Wang et al., 2003; Peng et al., 2010; Valderruten et al., 2014; Guo et al., 2016) were also detected in CS-SA. Differently, the peak at 1631 cm^−1^ assigned to the C=O of acid amide (Rao et al., 2012) in CS-SA showed more enhancement than CS, implying the increased acid amide group in CS-SA. It is worth noting that the strong peak at 1703 cm^−1^ assigned to the stretching vibration of carboxyl C=O in SA disappeared in CS-SA, and the N–H vibration peak at 1522 cm^−1^ in CS weakened in CS-SA, indicating the reaction sites located at –NH_2_ of CS and –COOH of SA. Therefore, the exact grafted reaction between SA and CS was confirmed based on ^1^H NMR and FT-IR.

### 3.2. Preparation and characterization of micelles

**Figure 4.**
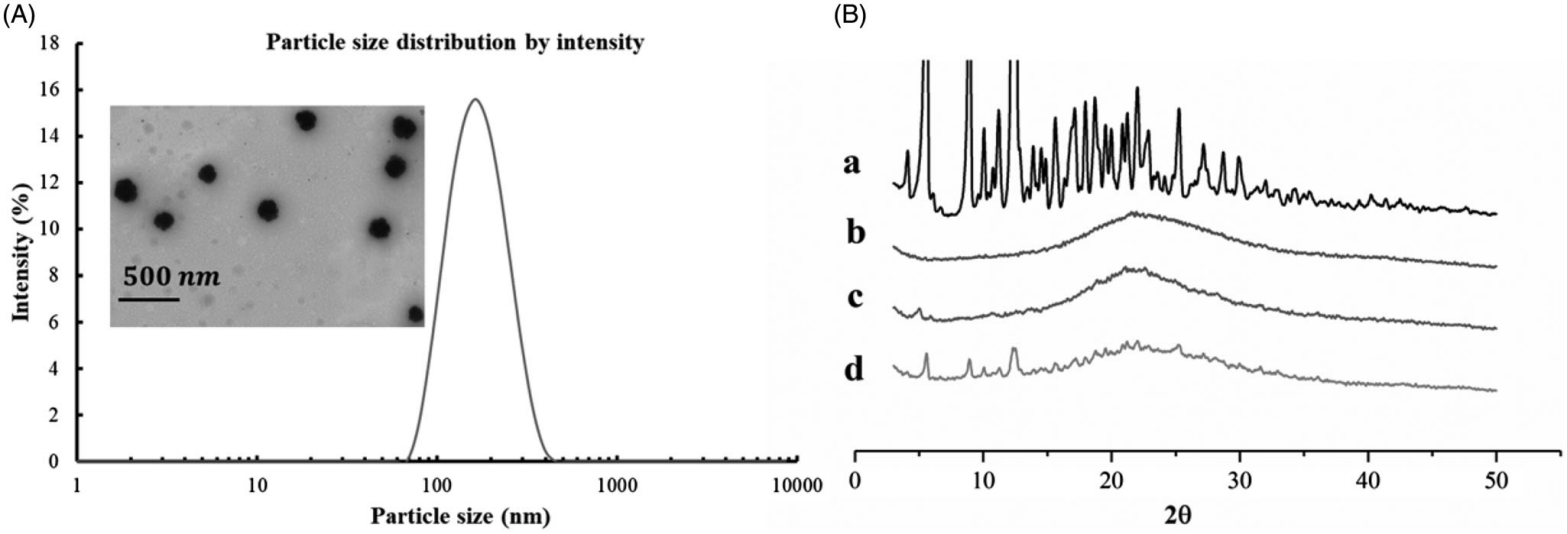
(A) Particle size distribution and TEM image of PTX-loaded LC-SA/CS-SA micelles; (B) the XRD curves of paclitaxel (a), PTX-loaded LC-SA/CS-SA micelles (b), PTX-free (blank) LC-SA/CS-SA (c), and physical mixture containing PTX and lyophilized blank LC-SA/CS-SA micelles (d).

The model drug PTX was successfully loaded into the LC-SA/CS-SA micelles by solvent evaporation-hydration method. Although the drug-free micelle can be prepared by simple self-assembly, the preparation of drug-loaded micelle must be well designed in view of the micelle particle size and drug-loaded capacity. As shown in Figure 4(A), the mean particle size of drug-loaded micelles was lowered to 157.1 nm with a narrow distribution (polydispersity index, PDI < 0.15), indicating the formation of compact micelle cores due to the interaction between hydrophobic stearoyl group and PTX (Zhang et al., 2015). The LC-SA/CS-SA micelles possessed positive zeta potential of +22.7 mV due to numerous amino groups on the micelle surface. The positively charged particles could be easily adsorbed by negative intestinal epithelial cell membrane (Pack et al., 2005), thereby, improving the intestinal absorption of drug encapsulated into the micelles. Besides, the particle size and zeta potential of micelles in solution did not vary significantly (RSD < 6%) at room temperature within seven days, suggesting the acceptable stability of the colloidal solution.

The micelles exhibited excellent DL capability with DL content of 15.96 ± 0.20 wt% and EE of 83.81%±0.12 wt%, as an efficient delivery system for oral administration. The high loading capacity of LC-SA/CS-SA micelle can be attributed to the hydrophobic core in favor of the encapsulation of PTX. Compared to other drug nano-carrier, the considerable DL or EE of micelle would be more beneficial to the improvement of drug absorption.

The TEM image of LC-SA/CS-SA micelles is shown in Figure 4(A). The drug-loaded micelles manifested regularly spherical shapes and homogeneous dispersion without aggregation due to the positive micelle surface. The micrographic micelle sizes were smaller than that measured by particle size meter, owing to the shrinkage of micelle caused by the dry treatment of sample.

To evaluate the crystallization behavior of PTX in micelles, XRD was conducted to raw PTX, blank micelles, drug-loaded micelles, simple physical mixture containing PTX, and lyophilized blank LC-SA/CS-SA micelles, as shown in Figure 4(B). Obviously, the PTX raw material was highly crystalline, whereas the blank micelles were X-ray amorphous. The typical diffraction peaks of PTX were visible in the range from 5° to 25° (2*θ*), and they were also found in the pattern of the physical mixture containing PTX and blank LC-SA/CS-SA micelles. However, the typical crystal peaks cannot be observed in drug-loaded micelles, and only one wide band was left, indicating that the model drug PTX was encapsulated in the polymeric micelles in the form of molecular or amorphous state instead of free crystal drug.

### 3.3. Determination of CMC

**Figure 5.**
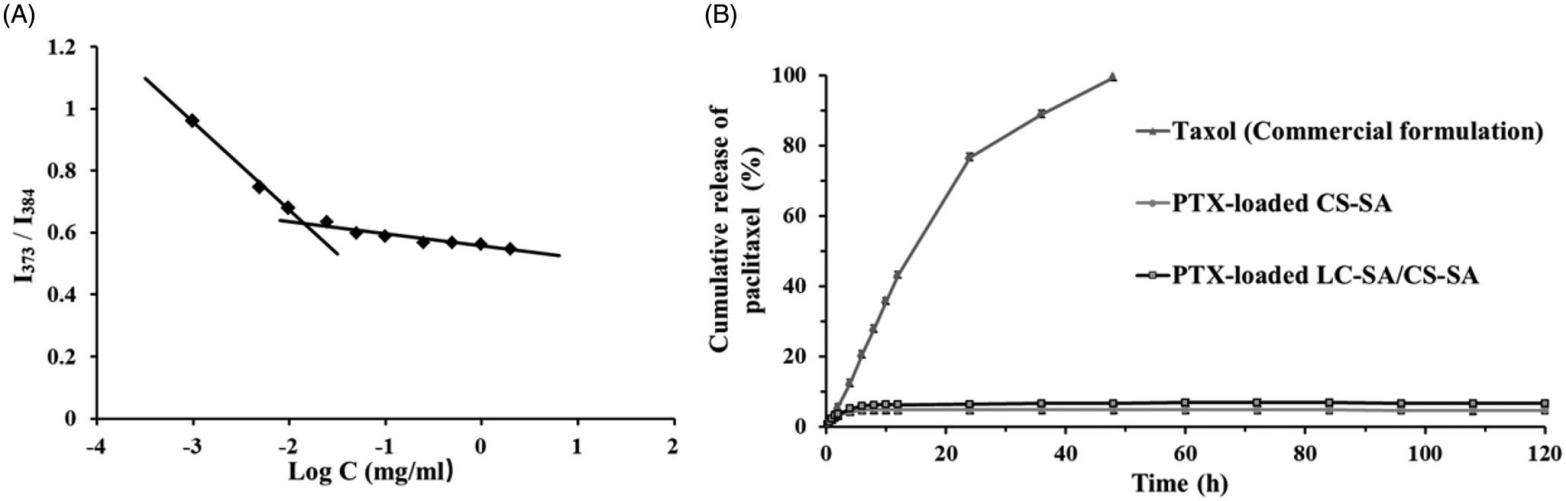
(A) The curve of the fluorescence intensity ratio (*I*_373_/*I*_384_) from pyrene vs. the logarithmic concentration of the material LC-SA/CS-SA; (B) the *in vitro* release profiles of Taxol (commercial formulation), PTX-loaded CS-SA micelles and PTX-loaded LC-SA/CS-SA micelles.

In the process of micellization, CMC was a key index to evaluate self-aggregation ability of micelles. The aggregation behavior of the material LC-SA/CS-SA in aqueous media was determined by fluorescence probe technique using pyrene as fluorescence probe (Shi et al., 2015). The curve of *I*_1_/*I*_3_ value vs. the logarithmic concentration of the micelle material LC-SA/CS-SA is shown in Figure 5(A). In the range of low concentration, the *I*_1_/*I*_3_ values were dropped sharply as there is increase in concentration, indicating that the polarity of solution was dramatically changed by the addition of the material. When the concentration increased to a certain value, the incremental velocity of the ratio became low, the transition concentration of variation tendency was defined as CMC, implying the rapid formation of micelles and the encapsulation of pyrene in micelles. The CMC value measured was low to 14.31 μg/ml, suggesting that the LC-SA/CS-SA micelles can keep their stability in solution, even under extreme dilution. Therefore, the micelle formulation can tolerate the dilution of gastrointestinal fluid and keep intact drug-loaded micelle form in intestinal tract for drug release.

### 3.4. *In vitro* release of paclitaxel-loaded micelles

The *in vitro* releases of PTX-loaded LC-SA/CS-SA micelles, PTX-loaded CS-SA micelles, and Taxol™, were carried out by dialysis against the release medium of phosphate buffer (pH 6.8, containing 2% Cremophor EL (w/v)). Compared to Taxol™, the LC-SA/CS-SA and CS-SA micelles showed significant difference in drug release, and a sustained drug release was observed with a biphasic trend, characterized as a relatively rapid initial phase, followed by a slower release phase. As illustrated in Figure 5(B), almost all the drugs were released from Taxol™ within 24 h; however, only 4.8% and 6.6% of PTX were released from CS-SA and LC-SA/CS-SA micelles, respectively within 120 h. The release profile of LC-SA/CS-SA micelles was very similar to CS-SA micelles, and only a small part of PTX in both micelles was released. The test result indicated that the LC-SA/CS-SA micelles could prevent drug leakage in intestinal tract before oral absorption. However, it was impossible for PTX to release into gastrointestinal fluid from the micelles before being absorbed.

### 3.5. Pharmacokinetic evaluation

**Figure 6.**
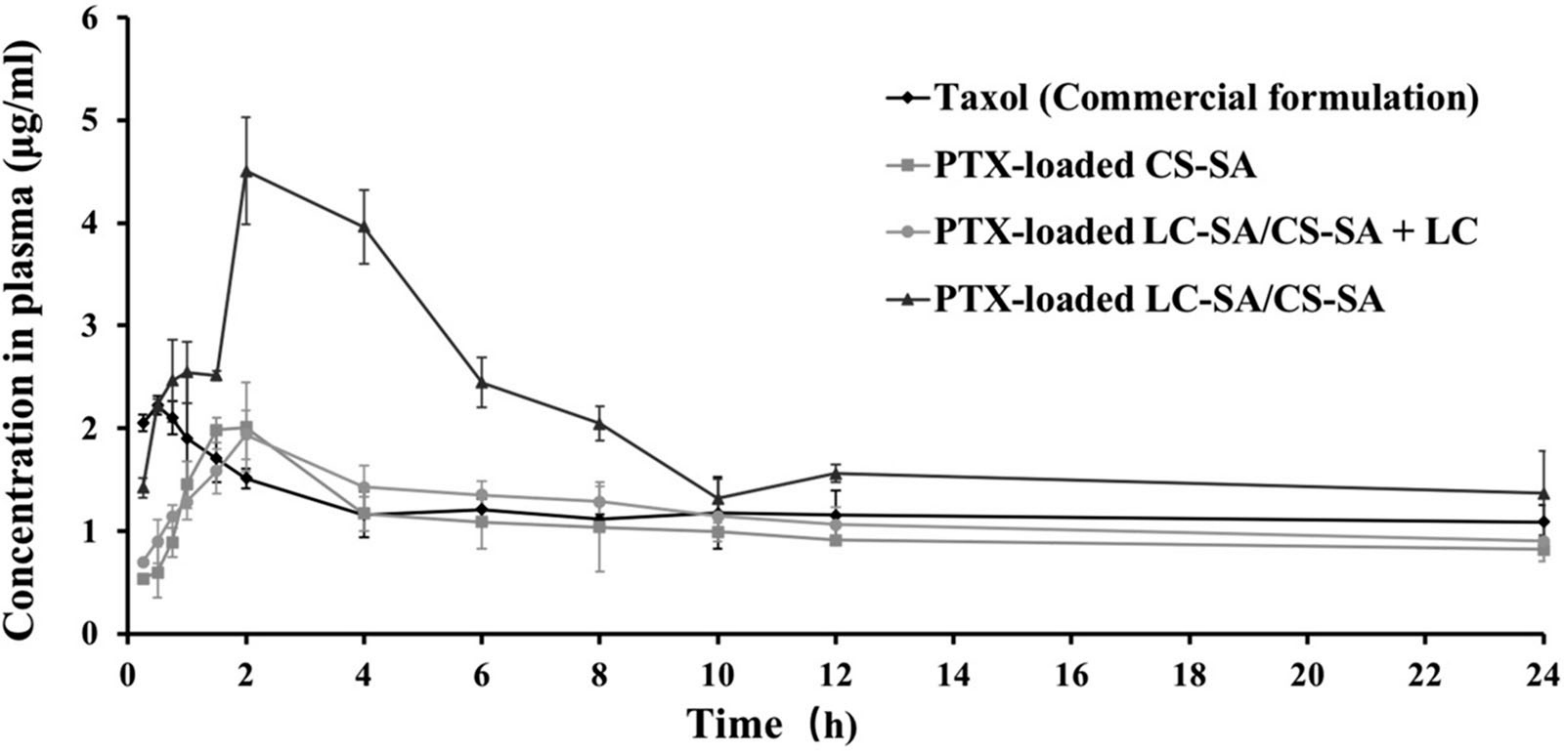
Oral pharmacokinetic profiles of Taxol^TM^ (commercial formulation), PTX-loaded CS-SA micelles, PTX-loaded LC-SA/CS-SA micelles plus LC, and PTX-loaded LC-SA/CS-SA micelles in rats as per the PTX-equivalent dose of 30 mg/kg. The data are expressed as means ± S.D. (*n* = 5).

The pharmacokinetic study of PTX-loaded LC-SA/CS-SA micelles was performed on SD rats, against Taxol™ and drug-loaded CS-SA micelles as reference formula. Since LC as the intrinsic ligand of OCTN2 can occupy the binding site of the transporter and competitively inhibit the transporter binding of LC-conjugated micelle, PTX-loaded LC-SA/CS-SA micelle plus LC with the equivalent molar concentration of LC-SA, as test formulation, was also investigated for the confirmation of drug absorption route. All the preparations were orally administrated as per the PTX-equivalent dose of 30 mg/kg, and the curves of the plasma concentration vs. time are shown in Figure 6 and the pharmacokinetic parameters are listed in Table 1. It was shown that all the preparations were rapidly absorbed within 2 h, and the plasma concentrations of LC-SA/CS-SA micelles were much higher than that of CS-SA micelles and Taxol™. Compared to the commercial formulation Taxol™, the LC-SA/CS-SA micelles showed 2.03-fold peak concentration (*C*_max_) and the relative bioavailability of 165.8%, indicating the enhanced absorption. The limited improvement of bioavailability suggested that the target delivery system still underwent the possible absorption obstruction from P-gp efflux and cytochrome P450 metabolism, which was known in drug oral absorption. Hence, the corresponding inhibitors may be the prospective synergistic promoters for the enhanced absorption of LC-SA/CS-SA micelles. There was no significant difference in AUC_(0–_*_t_*_)_ and *C*_max_ between Taxol™ and CS-SA micelle, implying the unmodified micelle lacked the ability of promoting drug absorption. It was worth noting that the additional LC in LC-SA/CS-SA micelle solution (PTX-loaded LC-SA/CS-SA + LC) brought about an absorption inhibition of PTX, and the pharmacokinetic profile and parameters were similar to the CS-SA micelles. The LC dissolved in the micelle solution, as a soluble ligand of OCTN2, was ready to target the transporter, hence the OCTN2 binding of LC-conjugated micelle was competitively inhibited. Therefore, the LC conjugation of CS-SA micelle was the key factor of enhanced absorption, and it can be confirmed that the improved oral bioavailability was caused by OCTN2-transporter on the basis of LC as a known ligand of the transporter. Interestingly, the LC-SA/CS-SA micelle showed obviously lower apparent volume of distribution (*V*_z_/*F*) than Taxol™ and CS-SA micelles, implying the possible target effect in circulation. Moreover, the significantly sustained *in vitro* release of the modified micelle surprisingly received higher *in vivo* bioavailability than the reference formula. All the facts pointed out a reasonable possibility that the PTX-loaded modified micelles were absorbed in the form of intact micelles instead of the foregone drug release in gastrointestinal tract before being absorbed, implying the drug absorption mechanism of OCTN2-mediated transportation. The modified micelles obtained shorter half-life (*t*_1/2z_) and higher plasma clearance (CLz/*F*), suggesting the fast clearance of the absorbed intact micelles from circulation (Zhao et al., 2016).

**Table 1.** Oral pharmacokinetic parameters of Taxol^TM^ (commercial formulation), PTX-loaded CS-SA micelles, PTX-loaded LC-SA/CS-SA micelles plus LC, and PTX-loaded LC-SA/CS-SA micelles in rats.

Parameters	Taxol™	PTX-loaded CS-SA	PTX-loaded LC-SA/ CS-SA + LC	PTX-loaded LC-SA/ CS-SA
AUC_(0–_*_t_*_)_ (mg/L·h)	28.98	24.43	28.24	48.05
*t*_1/2z_	83.83	44.85	30.78	9.40
*T*_max_ (h)	0.5	2	2	2
*C*_max_ (mg/L)	2.22	2.01	1.94	4.51
CLz/*F* (L/h/kg)	0.19	0.39	0.44	0.53
*V*_z_/*F* (L/kg)	23.36	25.09	19.53	7.17
Relative bioavailability (%)	–	84.30	97.45	165.80

### 3.6. Cellular uptake studies

**Figure 7.**
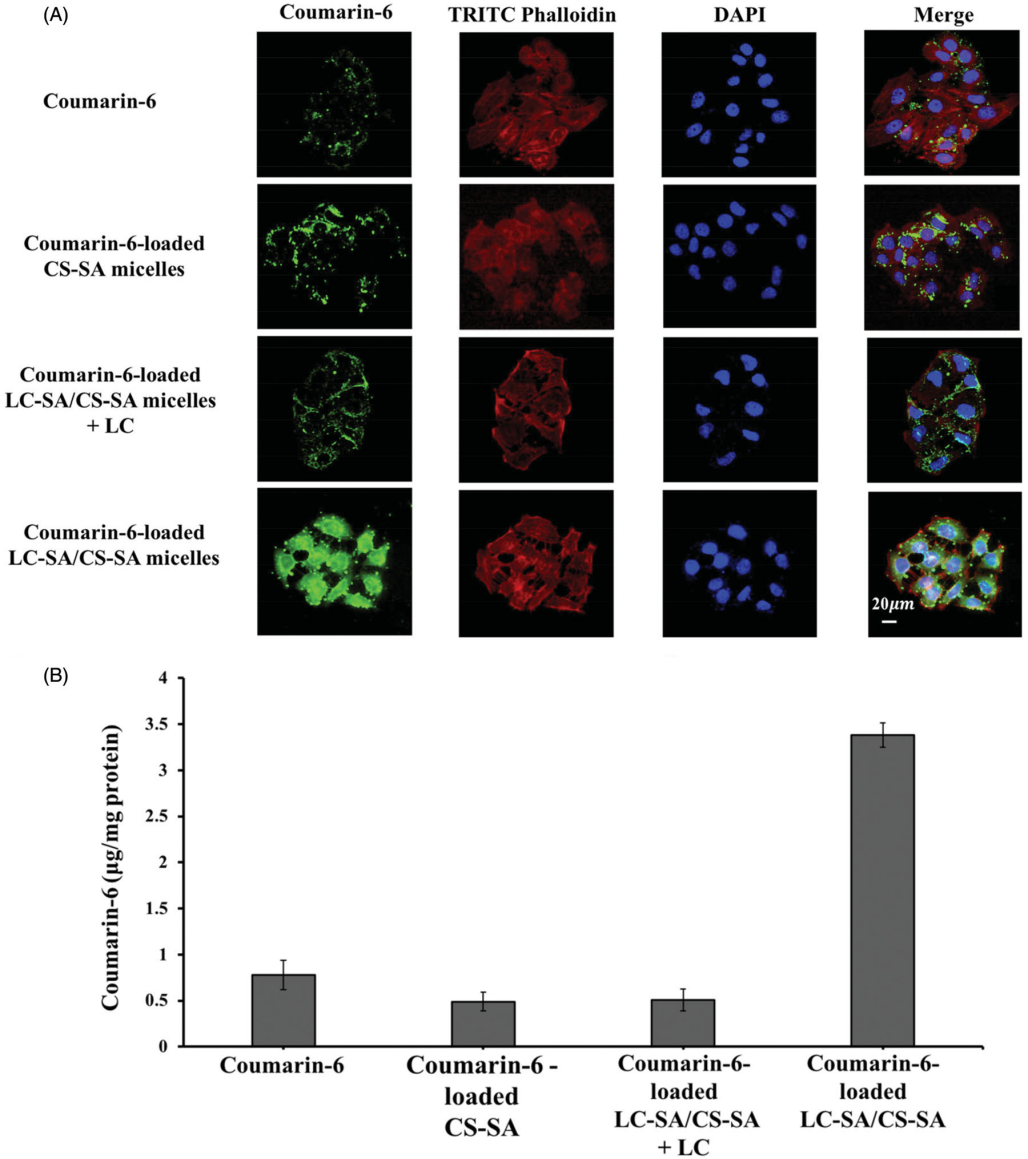
(A) Intracellular trafficking of coumarin-6, coumarin-6-loaded CS-SA micelles, coumarin-6-loaded LC-SA/CS-SA micelles plus LC, and LC-SA/CS-SA micelles after incubation for 1.5 h at 37 °C in Caco-2 cells. The cytoplasm (F-actins) was stained with TRITC-Phalloidin (red) and the nucleus with DAPI (blue), and the green fluorescence came from coumarin-6; (B) the quantitative uptake histogram of coumarin-6, coumarin-6-loaded CS-SA micelles, coumarin-6-loaded LC-SA/CS-SA micelles plus LC, and LC-SA/CS-SA micelles in Caco-2 cells. Data are shown as mean ± S.D. (*n* = 3, *p*< .05).

The fluorescence microscopy images of the cellular uptake effect in Caco-2 cells are shown in Figure 7(A). The uptake level of coumarin-6-loaded LC-SA/CS-SA micelles was obviously more enhanced than the reference coumarin-6 and coumarin-6-loaded CS-SA micelles, judged by the green fluorescence intensity comparison of coumarin-6, indicating that the optimized LC-SA/CS-SA micelles manifested significantly higher cellular uptake in Caco-2 cells. Interestingly, the coumarin-6 fluorescence of the LC-SA/CS-SA micelles was weakened by the addition of LC (LC-SA/CS-SA micelles plus LC), suggesting the key role of OCTN2 in the cellular uptake route. Therefore, the anchoring of LC-SA on the micelle surface facilitated its entrance into Caco-2 cells, owing to the transmembrane transport via OCTN2 expressed on the cell membrane. Furthermore, the coumarin-6 fluorescence of the modified micelles was distributed in the whole cell area including cytoplasm and nucleus, distinguished by the red TRITC-phalloidin fluorescence locating at cytoplasm and the blue DAPI at nucleus.

The quantitative results of cellular uptake are shown in Figure 7(B). The maximum amount of coumarin-6 absorbed in Caco-2 cells was still assigned to the LC-SA/CS-SA micelles by unit protein amount in cells, reaching to 3.38 μg per milligram protein. The uptake amount of the modified micelles was 4.33-fold and 7.04-fold higher than that of coumarin-6 and CS-SA micelles, respectively. The enhanced uptake effect of the target micelle was supported by both the fluorescent and quantitative measurements. Similar to the fluorescent result, the uptake amount of LC-SA/CS-SA micelles plus LC (0.508 μg per milligram protein) also manifested the competitive inhibition of LC and the OCTN2-mediated cellular uptake route of LC-conjugated micelle. Since the modification of LC on micelle surface promoted the cellular uptake of the encapsulated drug, it can be deduced that the LC-conjugated micelles entered into cells in the form of intact carriers via OCTN2 mediation. Although the bioadhesiveness of the micelle outer CS was favorable for drug absorption or uptake, there was no significant difference in the fluorescent and quantitative results between coumarin-6 and CS-SA micelles, in complete accord with the pharmacokinetic studies, suggesting that the enhanced effect of absorption was dependent on the ligand LC modification and OCTN2 transportation.

## 4. Conclusions

In this study, the LC-conjugated CS-SA micelles were successfully prepared and the model drug PTX was encapsulated in the polymeric micelles. The target carriers possessed a series of excellent physicochemical properties, including small particle size, positive zeta potential, high DL or EE, low CMC and delayed *in vitro* release, supporting the encapsulation and oral administration of water-insoluble drug. The animal and cellular studies suggested that the LC-SA/CS-SA micelles can be considered as a potential candidate for improving the oral bioavailability of poorly soluble drugs. The OCTN2-mediated transportation mechanism of the target micelle was supported by the present evidence, and the absorption mode of intact micelle can be reasoned out according to the pharmacokinetic and cellular uptake studies. The proposed delivery system would pave a way to enhance the therapeutic effect of oral drugs.
